# Antibiotic resistance and prevalence of Stenotrophomonas maltophilia isolated from clinical samples

**DOI:** 10.1099/acmi.0.001256.v3

**Published:** 2026-10-20

**Authors:** Mohammed Lahmer, Abderrazak Saddari, Said Ezrari, Mostafa Elouennass, Adil Maleb

**Affiliations:** 1 Laboratory of Microbiology, Faculty of Medicine and Pharmacy, University Mohammed the First, Oujda, Morocco; 2 Laboratory of Microbiology, Mohammed VI University Hospital, Oujda, Morocco; 3 Laboratory of Bioresources, Biotechnology, Ethnopharmacology and Health, Faculty of Sciences, University Mohammed the First, 60000, Oujda, Morocco; 4 Department of Bacteriology, Mohammed V Teaching Military Hospital, Rabat, Morocco; 5 Epidemiology and Bacterial Resistance Research Team/BIO-INOVA Centre, Faculty of Medicine and Pharmacy, University Mohammed V, Rabat, Morocco

**Keywords:** antimicrobial resistance, healthcare-associated infections, non-fermenting Gram-negative bacillus, *Stenotrophomonas maltophilia*

## Abstract

*Stenotrophomonas maltophilia* is an emerging non-fermenting Gram-negative bacillus increasingly implicated in healthcare-associated infections. Its remarkable ability to form biofilms on inert surfaces and invasive medical devices, together with its intrinsic and acquired resistance mechanisms, makes it a major therapeutic challenge in hospital settings. This study aimed to describe the epidemiological characteristics and antimicrobial susceptibility profile of *S. maltophilia* isolates recovered in our microbiology laboratory.

A retrospective study was conducted on clinical isolates of *S. maltophilia* obtained from various clinical specimens. The bacterial examination was performed according to standard microbiological guidelines

Over the study period, the prevalence of *S. maltophilia* isolates was 0.25% among all bacterial isolates (34 out of 13,959). Respiratory specimens were the most frequent source (23.53%, *n*=8), followed by blood cultures (17.65%, *n*=6). Most isolates originated from intensive care units (47.06%, *n*=16). Trimethoprim–sulfamethoxazole retained high efficacy with a susceptibility of 81.48%, whereas Minocycline demonstrated the highest activity, with a susceptibility rate of 100%. In contrast, the high resistance rates were observed for ceftazidime (61.90%). *S. maltophilia* represents an emerging nosocomial pathogen with significant therapeutic implications. Continuous monitoring of local resistance profiles, along with strengthened infection prevention measures and appropriate antibiotic use, is essential to limit the spread of resistant strains.

## Introduction

*Stenotrophomonas maltophilia* is a non-fermenting, strictly aerobic, Gram-negative bacillus belonging to the family *Lysobacteriaceae* [1]. This organism is ubiquitous in nature and has been isolated from soil, freshwater, wastewater and various plants. In hospital environments, the biofilm-forming ability of this bacterium promotes its adherence to inert surfaces and invasive medical devices, establishing a significant reservoir and representing a potential source of nosocomial infections [2–4]. This pathogen was previously regarded as having limited pathogenic potential; however, with the increasing prevalence of immunosuppression in the general population, it is now recognized as a significant nosocomial pathogen [1]. Over the past decades, the incidence of *S. maltophilia* infections has increased, largely due to the growing use of invasive procedures, the widespread administration of broad-spectrum antibiotics and advances in microbiological identification techniques. Consequently, it has emerged as the most prevalent non-fermenting Gram-negative bacillus in clinical laboratories after *Pseudomonas aeruginosa* and *Acinetobacter baumannii* [5, 6]. Clinical manifestations are heterogeneous and predominantly include ventilator-associated pneumonia, intravascular catheter-related bacteraemia and chronic respiratory infections in patients with cystic fibrosis [2, 6]. The considerable genetic plasticity of this pathogen, combined with its ability to form biofilms, its inherent resistance to numerous antibiotics, and the emergence of resistant strains, renders it a major therapeutic concern [4]. Accordingly, knowledge of local epidemiology and resistance trends is crucial for tailoring empirical antimicrobial therapy and implementing appropriate preventive strategies. This study aims to characterize the epidemiological features and antibiotic susceptibility patterns of *S. maltophilia* isolates from our microbiology laboratory.

## Methods

This is a retrospective study conducted at the microbiology laboratory of University Hospital, covering 63 months from January 2021 to May 2026. It includes all samples positive for *S. maltophilia* among those received for cytobacteriological examination. Specimens were transported and processed rapidly in accordance with standard microbiological guidelines [7]. Matrix-assisted laser desorption/ionization time-of-flight mass spectrometry and automated biochemical identification systems (BD Phoenix) were used for bacterial identification. Antimicrobial susceptibility testing was carried out using two complementary approaches: automated determination of the minimum inhibitory concentration using the BD Phoenix system and the disc diffusion method on Mueller–Hinton agar. Antimicrobial susceptibility results were interpreted according to the European Committee on Antimicrobial Susceptibility Testing clinical breakpoints and recommendations applicable at the time each isolate was recovered [8].

Data were extracted from the laboratory information management system (HOSIX). For each patient, the following variables were collected: age, sex, hospital department, type of specimen, identified bacterial species and antimicrobial susceptibility profiles. Statistical analysis was performed using Microsoft Excel 2019 software.

## Results

Over the study period, 34 *S. maltophilia* isolates were collected, corresponding to a prevalence of 0.25% among all bacterial isolates (34 out of 13,959). Mal was predominant, accounting for 64.71% (*n*=22), yielding a male-to-female ratio of 1.84. The mean age of the study population was 36.55 years. The most represented age group was 0–15 years (26.47%, *n*=9), followed by 30–45 years (23.53%, *n*=8). Regarding specimen type, the respiratory sample was the most frequent source (23.53%, *n*=8), followed by blood cultures (17.65%, *n*=6). Concerning hospital departments, the highest proportion of isolates originated from intensive care (47.06%, *n*=16) (Table 1).

**Table 1.** Demographic and clinical features of the study population

Characteristics	Number	Percentage
Gender		
Women	12	35.29%
Men	22	64.71%
Age group		
0–15	9	26.47%
15–30	5	14.71%
30–45	8	23.53%
45–60	4	11.76%
60–75	6	17.65%
75–90	2	5.88%
Department		
Intensive care	16	47.06%
Burn unit	2	5.88%
Thoracic surgery	1	2.94%
Internal medicine	2	5.88%
Nephrology	3	8.82%
Ophthalmology	1	2.94%
Otorhinolaryngology	1	2.94%
Paediatric	3	8.82%
Externe	2	5.88%
Traumatology	3	8.82%
Specimen type		
Intravascular device	3	8.82%
Urine	1	2.94%
Blood culture	6	17.65%
Ascitic fluid	1	2.94%
Pleural fluid	3	8.82%
Deep pus	4	11.76%
Superficial pus	8	23.53%
Protected distal bronchial specimen	3	8.82%
Sputum culture	1	2.94%
Bronchial aspirate	4	11.76%

The antimicrobial susceptibility profile revealed marked differences among the tested agents. Minocycline demonstrated the highest activity, with a susceptibility rate of 100%. In contrast, the highest resistance rate was observed for ceftazidime (61.90%) (Fig. 1).

**Fig. 1.**
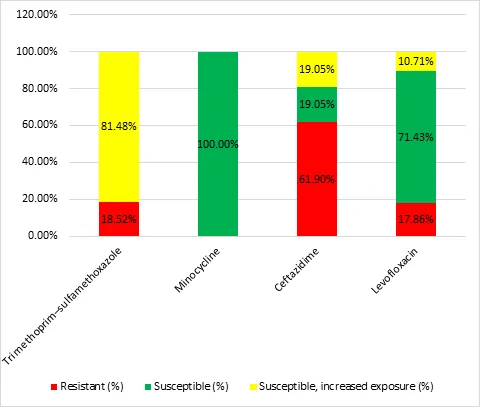
Antimicrobial resistance profile of our isolates.

## Discussion

Non-fermenting Gram-negative bacilli, including *P. aeruginosa*, *A. baumannii*, *S. maltophilia* and *Burkholderia cepacia*, pose a significant therapeutic challenge owing to their inherent resistance to multiple classes of antibiotics and their ability to develop additional resistance mechanisms under antimicrobial pressure [9]. Within this group, *S. maltophilia* has progressively emerged as a significant opportunistic pathogen, causing severe healthcare-associated infections. The intrinsic resistance of this organism to β-lactams and a wide range of other antibiotics greatly restricts treatment options and poses serious challenges for clinical management [10]. Accordingly, investigating the prevalence and antimicrobial resistance patterns of *S. maltophilia* within our hospital setting may provide critical insights for informing the effective management of infections caused by this pathogen.

Over the study period, the prevalence of *S. maltophilia* isolates was 0.25% among all bacterial isolates (34 out of 13,959). The global prevalence of *S. maltophilia* infections varies according to geographic region, type of healthcare facility and the populations studied. Most reported cases have been documented in European, Asian and American countries, whereas no relevant studies have been conducted on the African continent. International data indicate that *S. maltophilia* has become one of the most common non-fermenting Gram-negative bacilli, with a gradual increase in its isolation rates, particularly in intensive care units (ICUs). A recent meta-analysis based on the World Health Organization (WHO) regional classification demonstrated marked geographical variation in the prevalence of *S. maltophilia* infections. The highest prevalence was reported in the Western Pacific Region (10.5%), followed by the European Region (7.9%), whereas the lowest prevalence was observed in the Region of the Americas (4.3%) [11]. Similarly, data from the 2021 CHINET bacterial resistance surveillance programme showed that *S. maltophilia* represented 2.78% of all bacterial isolates and ranked as the third most frequently isolated non-fermenting Gram-negative bacillus [12]. Our isolates were predominantly obtained from ICUs. This finding is consistent with several published series reporting an increased detection of *S. maltophilia* in ICU settings [13–16]. Several factors may account for this predominance, including the frequent use of invasive devices such as mechanical ventilation and central venous catheters, prolonged hospital stays and extensive exposure to broad-spectrum antibiotics. Together, these conditions favour both colonization and infection with *S. maltophilia*, while also contributing to the emergence of resistant strains [5, 17]. Respiratory tract infections were the most commonly reported infections in our series, especially among patients receiving mechanical ventilation [18]. Indeed, several recent studies investigating the epidemiological and clinical characteristics of *S. maltophilia* have identified respiratory specimens as the most common source of isolation, followed by blood samples [11, 18, 19]. Similarly, data from the 2024 Spanish national surveillance programme for ICU-acquired infections showed that *S. maltophilia* accounted for 2.9% of all isolated micro-organisms, was mainly implicated in ventilator-associated pneumonia (5.81%) and ranked as the fifth most frequently isolated pathogen after *P. aeruginosa*, *Klebsiella pneumoniae*, *Staphylococcus aureus* and *Serratia marcescens* [20]. The bacterium’s ability to adhere to plastic surfaces and form a biofilm on hospital equipment, such as ventilation circuits and medical devices, explains its high prevalence in the above-mentioned samples [5, 11]. *S. maltophilia* exhibits resistance mechanisms to most antibiotics. It possesses two inducible chromosomal genes, L1 (a metallo-β-lactamase) and L2 (a serine β-lactamase), which hydrolyse all β-lactams, including carbapenems and aztreonam. Moreover, it is intrinsically resistant to aminoglycosides through the production of proteases and modifying enzymes. The presence of efflux pumps could also compromise the activity of older tetracyclines and aminoglycosides. The existence of the chromosomal Smqnr gene further reduces the activity of fluoroquinolones. In addition, other genes may be acquired, conferring resistance to a wide range of agents, including trimethoprim–sulfamethoxazole (TMP–SMX). Consequently, this pathogen demonstrates high levels of both intrinsic and acquired resistance, which considerably restrict the available therapeutic options [2, 4, 6, 9, 21].

In our study, the resistance rate to ceftazidime was the highest, which is consistent with the expression of the bacterium’s intrinsic β-lactamases. Similarly, a study conducted by Taghreed A. Hafiz *et al*. in Saudi Arabia reported that *S. maltophilia* isolates were predominantly resistant to ceftazidime (62.1%) [17]. Furthermore, according to a recent meta-analysis, ceftazidime exhibited high resistance rates across all WHO-classified regions (Region of the Americas :56.4%; Eastern Mediterranean Region: 42.9%; South-East Asia Region: 65.1%; Western Pacific Region: 52.6%) [11]. Based on its high susceptibility rates, TMP–SXT is generally recommended and widely used for the treatment of infections caused by *S. maltophilia*. However, resistance to this agent has increased over the years, and the relatively frequent adverse drug reactions may further limit its clinical use [22]. The resistance rate to SXT among our isolates was 18.52%. These findings are consistent with those of a recent meta-analysis that reported a relatively high overall resistance rate to TMP–SMX in Africa [23]. However, data from the SENTRY Antimicrobial Surveillance Program reported a high susceptibility rate to TMP–SMX, reaching 95.7% [24]. Minocycline remains one of the most active molecules, with a low resistance rate observed, confirming its potential therapeutic value [11]. Several studies have shown that minocycline may be more appropriate in terms of sensitivity and could be the first-line treatment for *S. maltophilia* infections, particularly those resistant to TMP–SMX [22]. Thus, a recent systematic review showed that overall rates of resistance to minocycline were 1% [25]. Also, in Thailand, a study of hospitalized patients demonstrated that minocycline was the most effective against *S. maltophilia* isolates, with a sensitivity rate of 100% [26]. These data are compatible with our results, which showed 100% sensitivity to minocycline. TMP–SMX and minocycline are recommended by the Infectious Diseases Society of America committee as the first-line drugs for monotherapy treatment of mild infections and as part of combined therapy for moderate to severe infections [27]. Fluoroquinolones represent an alternative therapeutic option due to their comparable efficacy to TMP–SMX, with fewer adverse effects. However, the rapid emergence of quinolone resistance, both *in vitro* and *in vivo*, when levofloxacin is used for the treatment of *S. maltophilia* infections remains a significant concern [11, 28]. In our study, 17.86% of the isolates were resistant to levofloxacin. A recent systematic review reported an overall levofloxacin resistance rate of 14%, with significant variations across geographic regions. However, a cohort study conducted in hospitals in Japan reported a relatively higher levofloxacin resistance rate (35%) [28]. Several novel antimicrobial agents may represent promising alternatives for the treatment of *S. maltophilia* infections; however, their efficacy has not yet been tested in randomized clinical trials or real-world studies. Among these, cefiderocol has been approved for the treatment of infections caused by aerobic Gram-negative organisms in adults with limited treatment options. *In vitro*, this drug has shown 100% efficacy against *S. maltophilia* strains [29]. In addition, the combination of aztreonam with avibactam may constitute another potential therapeutic alternative, restoring susceptibility to aztreonam in up to 98% of cases [30].

## Conclusion

The increasing emergence of *S. maltophilia* as an opportunistic pathogen responsible for healthcare-associated infections is closely associated with the growing selective pressure exerted by antibiotics within hospital settings. To address this issue, the treatment of all infections should be guided by antimicrobial susceptibility testing to ensure the selection of the most appropriate therapeutic agent. Our findings suggest that TMP–SMX and minocycline may be considered first-line treatment options. Effective strategies to limit the spread of *S. maltophilia* rely on a combined approach that involves healthcare environment management, prevention of cross-transmission and judicious use of antibiotics and require close collaboration among infection control teams, microbiologists and infectious disease specialists.

## Abbreviations

ICU: intensive care unit
SXT: sulfamethoxazole
TMP: trimethoprim
WHO: World Health Organization
